# Integrated pan-cancer analysis and experimental verification of the roles of tropomyosin 4 in gastric cancer

**DOI:** 10.3389/fimmu.2023.1148056

**Published:** 2023-03-13

**Authors:** Qijing Guo, Linglin Zhao, Nan Yan, Yan Li, Cuiping Guo, Shengyan Dang, Xianliang Shen, Jianfang Han, Yushuang Luo

**Affiliations:** ^1^ Research Center for High Altitude Medicine, Key Laboratory of High Altitude Medicine (Ministry of Education), Key Laboratory of Application and Foundation for High Altitude Medicine Research in Qinhai Province (Qinghai-Utah Joint Research Key Lab for High Altitude Medicine), Laboratory for High Altitude Medicine of Qinghai Province, Qinghai University, Xining, China; ^2^ Department of Oncology, Affiliated Hospital of Qinghai University, Xining, China

**Keywords:** tropomyosin 4, pan-cancer, gastric cancer, integrative analysis, immune infiltrating, experiment

## Abstract

**Objective:**

To investigate the function of tropomyosin 4 (TPM4) using pan-cancer data, especially in gastric cancer (GC), using comprehensive bioinformatics analysis and molecular experiments.

**Methods:**

We used UCSC Xena, The Cancer Genome Atlas (TCGA), Genotype-Tissue Expression Project (GTEx), TIMER2.0, GEPIA, cBioPortal, Xiantao tool, and UALCAN websites and databases for the extraction of pan-cancer data on TPM4. TPM4 expression was investigated with respect to prognosis, genetic alterations, epigenetic alterations, and immune infiltration. RNA22, miRWalk, miRDB, Starbase 2.0, and Cytoscape were used for identifying and constructing the regulatory networks of lncRNAs, miRNAs, and TPM4 in GC. Data from GSCALite, drug bank databases, and Connectivity Map (CMap) were used to analyze the sensitivity of drugs dependent on TPM4 expression. Gene Ontology (GO), enrichment analyses of the Kyoto Encyclopedia of Genes and Genomes (KEGG), wound healing assays, and (Matrigel) transwell experiments were used to investigate the biological functions of TPM4 in GC.

**Result:**

The findings of the comprehensive pan-cancer analysis revealed that TPM4 has a certain diagnostic and prognosis value in most cancers. Alterations in the expression of TPM4, including duplications and deep mutations, and epigenetic alterations revealed that TPM4 expression is related to the expression of DNA methylation inhibitors and RNA methylation regulators at high concentrations. Besides, TPM4 expression was found to correlate with immune cell infiltration, immune checkpoint (ICP) gene expression, the tumor mutational burden (TMB), and microsatellite instability (MSI). Neoantigens (NEO) were also found to influence its response to immunotherapy. A lncRNA-miRNA -TPM4 network was found to regulate GC development and progression. TPM4 expression was related to docetaxel,5-fluorouracil, and eight small molecular targeted drugs sensitivity. Gene function enrichment analyses revealed that genes that were co-expressed with TPM4 were enriched within the extracellular matrix (ECM)-related pathways. Wound-healing and (Matrigel) transwell assays revealed that TPM4 promotes cell migration and invasion. TPM4, as an oncogene, plays a biological role, perhaps *via* ECM remodeling in GC.

**Conclusions:**

TPM4 is a prospective marker for the diagnosis, treatment outcome, immunology, chemotherapy, and small molecular drugs targeted for pan-cancer treatment, including GC treatment. The lncRNA-miRNA-TPM4network regulates the mechanism underlying GC progression. TPM4 may facilitate the invasion and migration of GC cells, possibly through ECM remodeling.

## Introduction

Globally, GC is ranked the fifth according to its incidence rate and third in terms of its mortality rate among cancers. Early-stage tumors can be resected endoscopically and through radical GC surgery. Nevertheless, because of the highly aggressive nature of GC, when diagnosed, the majority of patients with GC exhibit advanced GC progression and are likely undergoing treatment with fluorouracil-and platinum-based chemotherapy (1). Molecular targeted drugs, such as trastuzumab (anti-human epidermal growth factor receptor-2, HER-2) (2) and immunotherapy with nivolumab (anti-programmed cell death protein 1, PD-1), have gradually improved the prognosis of patients (3), but not in all cases. Therefore, identifying new prognostic biomarkers and molecular target (4)s are urgent to predict the prognosis of GC patients and guide individualized treatment.

Tropomyosin (TPM) is an actin-binding protein that maintains the stability of non-muscle cells and contraction of muscle cells (5). Reportedly, it is involved in the proliferation of cells, migratory processes, biomechanics, vesicle trafficking, and glucose metabolism in pathophysiological processes (6). There are four tropomyosin (TPM) genes, namely TPM1, TPM2, TPM3, and TPM4, in mammals. In recent years, the abnormal expression of TPM4 was investigated in multiple malignancies, invasive breast cancer (7), colon cancer (8), glioma (9), hepatocellular carcinoma (10), lung cancer (11), and ovarian cancer (12). TPM4 is a crucial intermediary in different human malignancies, but the association between the function of TPM4 and GC is currently unclear.

Bioinformatics analyses have revealed that TPM1 and TPM2 are potential diagnostic markers for bladder cancer. TPM4 influences the immune infiltration of Th1, macrophages, and neutrophils (13). TPM4 expression is of clinical significance, has prognostic value, and is related to immune infiltration in pancreatic cancer (14). TPM4 is upregulated and related to the malignant characteristics of gliomas, possibly *via* epithelial-mesenchymal transition (9). In our previous study, TPM4 was found to function as an oncogene that stimulates the proliferation of cells and prevents the death of cells by apoptosis both *in vitro* and *in vivo*. TPM4 was found to be expressed at higher levels in GC tissues than in paracancerous tissues (15). However, comprehensive studies on the relevance of TPM4 expression in tumor immune cell infiltration, ICP gene expression, TMB, MSI, NEO, ceRNA, drug sensitivity, and the pan-cancer mechanism of action of TPM4, especially in GC, have seldom been conducted. A comprehensive analysis of the pan-cancer function of TPM4 is necessary.

**Figure 1:**
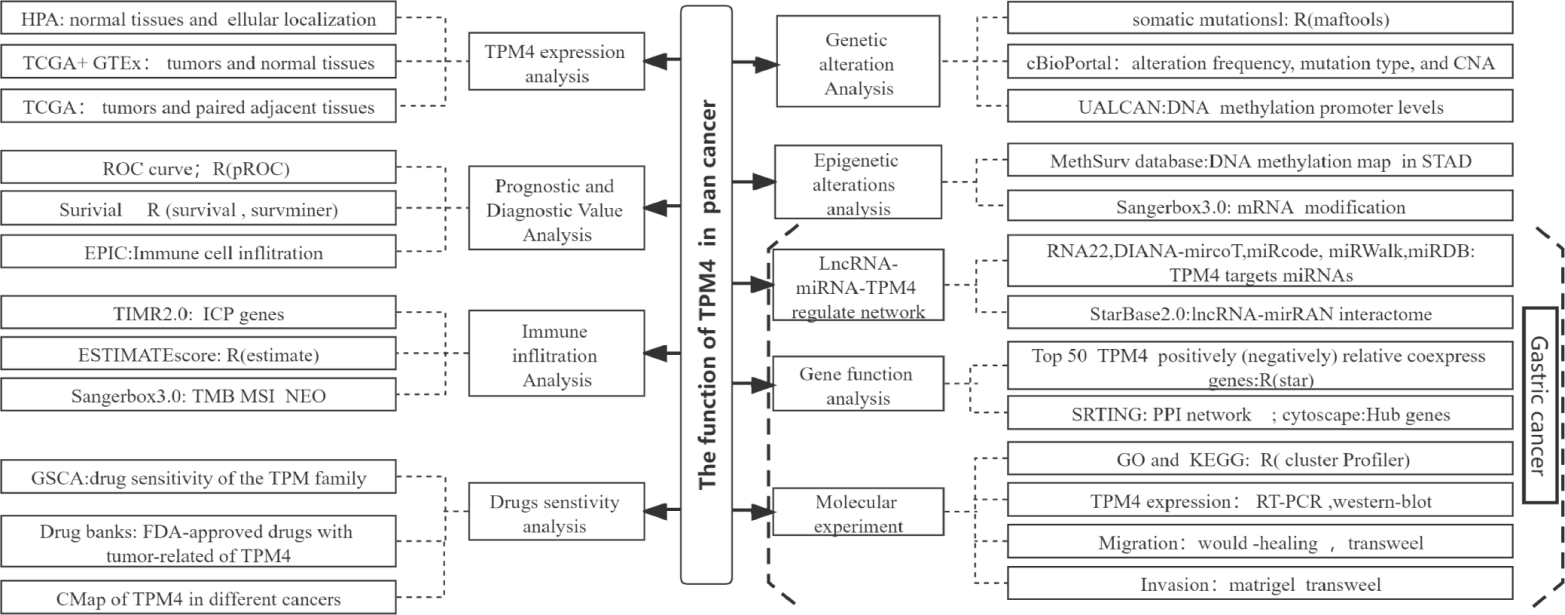
Workflow of the study.

We explored the expression pattern of TPM4 to determine its pan-cancer diagnostic and prognostic value, genetic changes, and epigenetic status. Additionally, we examined the relationship among the expression of TPM4, immune infiltration by pan-cancer cells, and the TPM4-associated antitumor drug response. Additionally, we explored the lncRNA-miRNA-TPM4 regulatory network in GC. TPM4 co-expression gene enrichment analysis was performed and validated in molecular experiments on GC cell lines. Our study is the first to reveal the potential applications of TPM4 as a predictive target for diagnosis, prognosis, as well as anti-cancer therapy within GC. We also revealed the potential role of TPM4 in promoting migration and invasion through extracellular matrix remodeling in GC (**Figure 1**, workflow of our study).

## Materials and methods

### Analysis of TPM4 expression and its subcellular localization

We used the human protein atlas (HPA, https://www.proteinatlas.org/) to examine TPM4 mRNA expression in normal tissues (N= 13,084) across Genotype-Tissue Expression (GTEx https://gtexportal.org/) (16). TPM4 mRNA expression in normal and tumor tissues (N= 15,776), the expression of TPM4 within tumors as well as within paired normal tissues (n=15043) and data from UCSC XENA (https://xenabrowser.net/datapages/) were used. We used GEPIA2’s “Stage Plot” module to evaluate the correlation between TPM4 expression, which was upregulated, and the pathological stages of cancers. To perform the analysis and comparison, RNAseq data from TCGA and GTEx were processed uniformly using the Toil process (17) and then log2-transformed. Xiantao tool (https://www.xiantao.love/ is a useful bioinformatics analysis web tool, and was used for visualization. Statistical analysis was performed using the Wilcoxon rank-sum test, and significant outcomes were defined at p < 0.05. We used the immunofluorescence staining images of three human cancer cell lines (A-431, U251MG, and U-2 OS) to display the subcellular localization of TPM4 in cancer cells from the HPA dataset.

### Analysis of the prognostic and diagnostic value of TPM4

We assessed variations within TPM4 expression for the diagnosis and prognosis of cancer using RNA sequencing data obtained from TCGA (https://portal.gdc.cancer.gov/). Xiantao tool was analyzed statistically using the log-rank test, with a P-value < 0.05 regarded as significant. We obtained the ''hazard ratio (HR) 95% CI'' as well as the ''P-value'' and used Xiantao tool to visualize the forest plot. The receiver operating characteristic (ROC) curve and area under the ROC curve were applied to evaluate the diagnostic value of TPM4 in pan-cancer tissues using Xiantao tool . Diagnostic value: low accuracy (AUC: 0.5–0.7), certain accuracy (AUC: 0.7–0.9), and high accuracy (AUC > 0.9).

### Genetic alteration analysis

cBioPortal (18) (http://www.cbioportal.org) provides a platform for analyzing and interpreting cancer genetic data and facilitates the interpretation of molecular data acquired from cancer histological and cytological studies. Gene alteration data from 2683 samples collected from 2565 pan-cancer patients obtained from UCSC Xena and the International Cancer Genome Consortium(ICGC) (https://www.icgc-argo.org) data portal from "TCGA pan-cancer Atlas Studies" were used for analysis. The mutation landscape of TPM4, including the mutation type, copy number alteration(CAN), as well as mutation frequency data, was searched using the module titled "Cancer Types Summary". Somatic mutation datasets from the publicly available TCGA database were acquired *via* the data portal to identify the genomic data commons (https://portal.gdc.cancer.gov/). The dataset includes data from patients with stomach adenocarcinoma (STAD) within the TCGA database with high TPM4 expression (n=212) and low TPM4 expression (n=21); these data were used with HOME for research, a useful online bioinformatic tool (https://www.home-for-researchers.com).

### DNA methylation and mRNA modification

UALCAN (http://ualcan.path.uab.edu/analysis.html) (19) was used to explore the promoter DNA methylation levels in TPM4 in normal and pan-cancer tissues. The beta value represents the DNA methylation level, hypomethylation (beta: 0.3–0.25), and hypermethylation (beta: 0.7–0.5) (20). The DNA methylation map of TPM4 in STAD was obtained from the MethSurv database (21) ("Gene visualization" module). mRNA modification analysis of 45 methylation regulators involved in n1-methyladenosine (m1A) and 5-methylcytosine (m5C) n6-methyladenosine (m6A) addition in pan-cancer tissues across TCGA was performed using SangerBox3.0, a helpful bio information online tool ("pan-cancer analysis-Mrna modification" module) (http://sangerbox.com/).

### TPM4 expression and immune correlation

The European Prospective Investigation into Cancer and Nutrition (EPIC) (22) (https://gfellerlab.shinyapps.io/EPIC_1-1/) is an online platform that provides the infiltration ratio of eight types of immune cells according to the expression information Scores for stromal, immune, and ESTIMATE cells within the TCGA. The STAD dataset was derived using the ''estimate'' (23) R package, which is used to estimate the tumor purity scores. TIMR2.0 (24) (http://timer.comp-genomics.org/) serves as a platform for analyzing the immunological characteristics of cancer in a systematic manner across TCGA. To determine the association with TPM4 expression as well as eight immune checkpoints, a module named "Gene_Corr" was employed. After we extracted the "P-value" and "r-value," we used the Xiantao tool to visualize using a heatmap. Relationships between TPM4 expression and the MSI, TMB, and NEO were obtained using SangerBox3.0 ("pan-cancer analysis - heterogeneity analysis" module).

### LncRNA-miRNA-TPM4 regulatory network construction

Five online prediction databases for miRNAs, namely RNA22 (http://cbcsrv.watson.ibm.com/rna22.html/) (25), DIANA-mircoT (http://diana.imis.athena-innovation.gr/DianaTools/index.php?r=microT_CDS/index) (26), miRcode (http://www.mircode.org/index.php) (27), miRWalk (http://mirwalk.umm.uni-heidelberg.de/) (28), and miRDB (http://mirdb.org/miRDB/) (29) were used for predicting the TPM4 target miRNAs. miRNAs that were retrieved in at least three databases were defined as target miRNAs. StarBase2.0 (https://starbase.sysu.edu.cn/) was used for analyzing the lncRNA-mirRAN interactome and the relevance of miRNA and TPM4 (lncRNA). ''Mammals, humans, hg19, strict stringency (≥5) of CLIP-Data, including or excluding Degradome-Data'' was used as the screening criteria. A Sankey diagram of the miRNA-lncRNA interactome and lncnRNA-miRNA-TPM4 network was visualized using the Xiantao tool and Cytoscape, respectively.

### Drugs response analysis

GSCALite (http://bioinfo.life.hust.edu.cn/web/GSCALite/) (30) is used for analyzing integrated mRNA expression, mutation, immune infiltration, methylation across TCGA datasets, and drug resistance datasets from GDSC (https://www.cancerrxgene.org/), and CRTP (http://portals.broadinstitute.org/ctrp/). We analyzed the drug sensitivity of the TPM family (TPM1, TPM2, TPM3, and TPM4) using data from GDSA and CRTP. According to the retrieved datasets, Food and Drug Administration (FDA)-approved chemotherapy drugs related to TPM4 expression were found in drug banks (https://go.drugbank.com/drugs). The outcomes were visualized with Xiantao tool ." Connectivity Map (CMap) (31) is an expression profiling database based on the expression of intervening genes to reveal functional links between small molecule compounds, genes, and disease states. Xiantao tool was used to identify the top 100 up-regulated and down-regulated differentially expressed genes (DEGs) between the TPM4 high-expression and low-expression groups. These DEGs were applied to querying against CMap to predict small molecular potential therapeutic drugs for cancer patients. Drugs with positive/ negative connectivity scores can induce/reverse effects against the input signature in human cell lines.

### Genes co-expressed with TPM4 and functional analysis

The top 300 genes showing positive relative co-expression and top 300 genes showing negative relative co-expression with TPM4 (|cor|>0.3, P below 0.05) were identified from TCGA using Xiantao tool for visualizing the heatmaps. The PPI network of the top 100 genes showing positive relative co-expression with TPM4 was identified using STRING (https://cn.string-db.org/), which lists publicly available PPI data (32). Hub genes were analyzed using ''MOCODE'' and ''CytoHubba'' in Cytoscape (edition 3.7.2). Enrichment analyses based on Gene Ontology and Kyoto Encyclopedia of Genes and Genomes were conducted for the top 300 genes showing positive relative co-expression with TPM4 using the Xiantao tool " intended for cluster information analysis.

### Cell lines, culture, and transfection

AGS and BGC-823 human GC cells were purchased from the Shanghai Cell Bank of the Chinese Academy of Sciences (Shanghai, China), cultured in RPMI 1640 medium supplemented with 10% fetal bovine serum (FBS) (Gibco, Grand Island, NY, USA) and 1% penicillin-streptomycin, and maintained at 37°C and 5% carbon dioxide. Recombinant lentiviral vectors for TPM4 RNAi (LV-TPM4) and lentiviral vectors for negative control (LV-Ctrl) were designed and packaged in 293T cells from GeneChem Co., Ltd. The TPM4 siRNA had the following sequence: 5′-GGAGGACAAATATGAAGAAGA-3′. The shTPM4/shCtrl cohorts had AGS/BGC-823 cells (5 × 103/well) subcultured in 96-well culture plates and infected with LV-TPM4/LV-Ctrl. The cells that were infected were selected by incubation with 2 µg/mL puromycin for 48 h. The efficiency for TPM4 knockdown was detected by western blotting.

### Real-time polymerase chain reaction (RT-PCR)

We performed RT-PCR to determine TPM4 mRNA expression in GC cells as well as the knockdown efficiency of the TPM4 TRIzol® Plus Purification Kit for RNA (12183-555; Invitrogen; Thermo Fisher Scientifc, Inc.). We referred to the kit instructions for generating reverse transcription cDNA and detecting PCR products in a fluorescent quantitative PCR instrument. GAPDH (glyceraldehyde-3-phosphate dehydrogenase) was used to be an internal control. The oligonucleotide primers indicated as follows were used for quantitative PCR: TPM4, 5'- TTGAGGAGGAGTTGGACAGGG-3' forward and 5'-CCAGGATGACCAGCTTACGAG-3' reverse; GAPDH, 5’-TGACTTCAACAGCGACACCCA-3' forward and 5’-CACCCTGTTGCTGTAGCCAAA-3’ reverse. The reaction conditions were as follows: 45 cycles, 95°C: pre-change for 15 s, 95°C: denaturation for 5 s, 60°C: annealing and extension for 30 s. The 2-ΔΔCt method was used to analyze the expression levels of each gene.

### Western blotting analysis

After the cells were digested with protein lysates, the total proteins of the AGS and BGC-823 cells were extracted. A BCA kit (Beyotime, P0010) was used for measuring the cellular protein content. 10% SDS-PAGE was used to separate different proteins, and 50 g of protein was loaded per lane. The proteins were transferred to polyvinylidene difluoride (PVDF) membranes and blocked with 5% milk. The primary rabbit antibodies used were anti-TPM4 (cat number: ab181085; 1:1,000; Abcam) and anti-GAPDH (cat number: sc-32233; 1:1,000; Santa Cruz). A processing film containing HRP-conjugated goat anti-rabbit IgG (cat. no. sc-2004; 1,000; Santa Cruz) and anti-mouse IgG (cat. no. sc-2005; 1,000; Santa Cruz) was used. The membranes were detected using an enhanced chemiluminescence detection system (Pierce; Thermo Fisher Scientific, Inc.) and visualized using the ChemiDoc system (BioRad Laboratories, Inc.). The intensity of the proteins was measured using ImageJ (edition 1.8.0; National Institutes of Health, Bethesda, MD, US).

### Wound-healing assay

AGS and BGC-823 cells (density 2.0 ×10^5^ cells/well) transfected with LV-TPM4/LV-Ctrl were subcultured in 6-well plates (37°C, 5% CO_2_ in an incubator), in a culture system of 100 μL/well for 24 h. The cells within the plate were scratched using a scratch tool. The serum-free medium was substituted, and images were acquired under a microscope (XDS-100, Cai Kang Optical Instrument Co, Ltd, China) at 0, 8, and 24 h.

### (Matrigel) Transwell assays

Transwell kits (cat.NO 2433 Corning, US) were applied. AGS and BGC-823 cells transfected with shTPM4 and shctrl were planted into the upper chamber (8 µm) at a density of 0.8 × 105 cells/well in a serum-free medium. For the transwell assay, the medium within the upper chamber was removed, 100 μL of serum-free medium was added, and 600 μL of 30% FBS medium was added for 16 h at 37°C. For the Matrigel transwell assay, 100 μL of serum-free medium was applied to the upper and lower chambers. A layer of matrigel matrix glue (Corning) (ratio of serum-free medium: matrix glue = 8:1) was coated within the lower chamber, and the cells were incubated for 24 h at 37 °C. Non-metastatic cells were removed from the chamber. The chamber was fixed with a 4% paraformaldehyde fixative for 30 min. Later, the cells were transferred by staining with 1% crystal solution on the membrane's lower surface for 1-3 min. The cells on the lower side of the membrane were counted. Images were recorded under a microscope (BX53, Olympus Company, Japan).

### Statistical analysis

Statistical analyses in this study were conducted using the above online database and ''R package (R studio edition: 1.2.1335, R edition: 3.6.3), as described above. GraphPad Prism 9.0 (GraphPad Software La Jolla, CA, USA) was used for the statistical analysis of experimental data. Differences were compared using a Student's *t*-test, and outcomes are shown as mean ± SD. Statistical significance was reported at *P<0.05, **P< 0.01, ***P <0.001, and ****P < 0.0001.

## Result

### TPM4 expression levels and its subcellular localization

**Figure 2:**
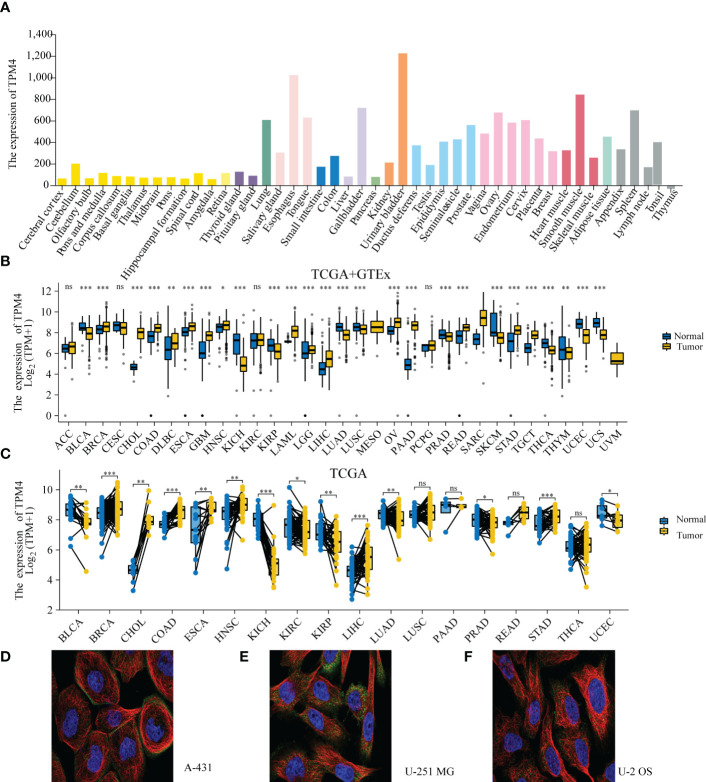
TPM4 expression levels and localization. **(A)** Expression levels of TPM4 in normal tissues based on HPA (n=13084). **(B)** TPM4 mRNA expression between tumors and normal tissues from TCGA+ GTE (N= 15,776). **(C)** The expression of TPM4 in tumors and paired adjacent normal tissues from TCGA (n=15043). **(D-F)**. Subcellular localization of TPM4 in A-431 cells from HPA datasets **(D)**. U-251MG cell line **(E)**, U-2 OS cell line **(F)** (ns, P≥ 0.05; *P < 0.05; **P < 0.01; ***P < 0.001).

The HPA datasets showed that the top-ranked expression of TPM4 was within the urogenital, respiratory, and digestive systems (**Figure 2A** and **Supplementary Table 1**). The TCGA and GTEx datasets revealed that TPM4 was significantly upregulated (P < 0.05) in BRCA, CHOL, COAD, DLBC, ESCA, GBM, HNSC, LAML, LGG, LIHC, LUCS, OV, PAAD, PCPG, PEAD, READ, SARC, STAD, and TGCT. In contrast, TPM4 was downregulated (P < 0.05) in BLCA, KICH, KIRP, LUAD, PRAD, LUSC, SKCM, THCA, THYM, UCEC, and UCS (**Figure 2B** and **Supplementary Table 2**). GEPIA2's dataset indicated PAAD and SKCM were significantly associated with tumor stage (P<0.05, **Supplementary Figure 1**). According to the outcomes of the TCGA dataset analysis, TPM4 was differentially expressed in tumor tissues and paired normal tissues in BLCA, BRAC, CHOL, COAD, ESCA, HNSC, KICH, KIRC, KIRP, LIHC, LUAD, PRAD, STAD, and UCEC (**Figure 2C** and **Supplementary Table 3**). The subcellular localization of TPM4 to actin filaments (**Figure 2D**) and the cytosol (**Figures 2E, F**) was observed. Therefore, our outcomes revealed that TPM4 expression was upregulated in most tumors and was greater than that in paired (unpaired) normal tissues in STAD.

### Correlation of TPM4 expression and pan-cancer prognosis and diagnosis

**Figure 3:**
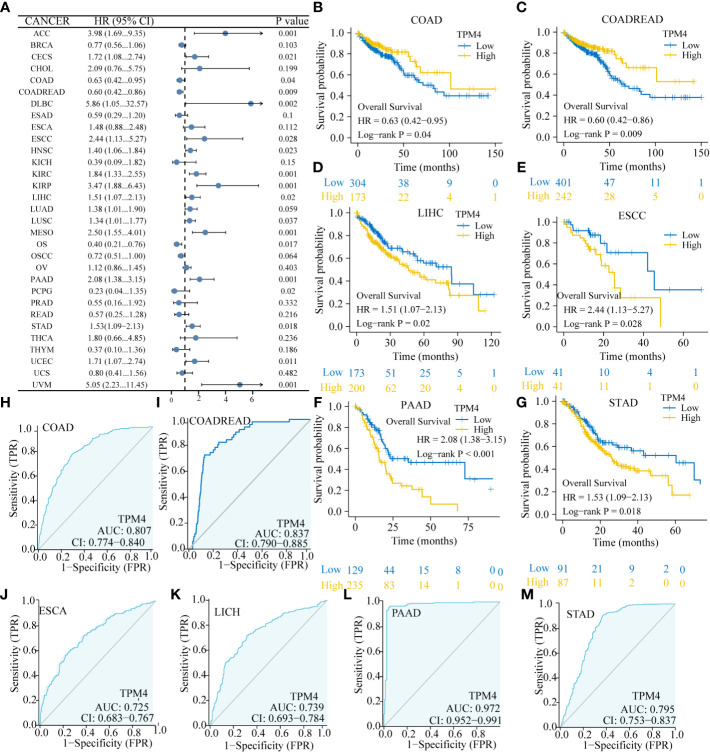
Correlation of TPM4 expression and pan-cancer prognosis and diagnosis. **(A)** Forest plot for the association between pan-cancer TPM4 expression and overall survival (p < 0.05). **(B-G)** Overall survival analyses for TPM4 expression in digestive system tumors. Patients with COAD ( (B ,n=521), COADREAD (C,n=698), LICH (D, n=424), ESCC (E,n=173), PAAD ((F,n=182), STAD (G,n=407). **(H-M)**. Diagnostic value of TPM4 in pan-cancer, as determined by ROC curve analysis. COAD ((H,n=521), COADREAD (I, n=698), ESCA ((J, n=173), LCHC (K,n=424) , PAAD ((L,n=182), STAD (M,n=407).

Based on the Forest plot and the findings of Cox assessment (**Figure 3A**), TPM4 is an adverse factor for overall survival (OS) in ACC, CESC, CHOL, DLBC, ESCC, HNSC, KIRC, KIRP, LIHC, LUAD, LUSC, MESO, PAAD, STAD, UCEC, and UVM (P < 0.05, HR > 1), whereas it acts as a potentially beneficial factor in COAD, COADREAD, OS, and PCPG (P < 0.05, HR < 1). Furthermore, we focused on the association involving TPM4 expression and digestive system malignancies, with a high expression of TPM4 linked significantly to poor prognosis in, LIHC (**Figure 3D**, p=0. 02), ESCC (**Figure 3E**, p=0.028), PAAD (**Figure 3F**, p < 0.001), and STAD (**Figure 3G**, p=0.018). Notably, patients with low TPM4 expression show worse prognosis than patients with high TPM4 expression in cases of COAD (**Figure 3B**, p=0.04) and COADREAD (**Figure 3C**, p=0.009). Next, we used ROC curves to assess the diagnostic efficacy of TPM4 in digestive cancers. TPM4 had a certain accuracy in predicting COAD (AUC = 0.807) (**Figure 3H**), COADREAD (AUC = 0.837) (**Figure 3I**), ESCA (AUC = 0.725) (**Figure 3J**), LICH (AUC = 0.739) (**Figure 3K**), and STAD (AUC = 0.795) (**Figure 3L**). TPM4 expression also had a high accuracy in predicting PAAD (AUC = 0.972) (**Figure 3M**). Collectively, TPM4 expression has diagnostic and prognostic value in different cancers, including STAD.

### Genetic alteration analysis

**Figure 4:**
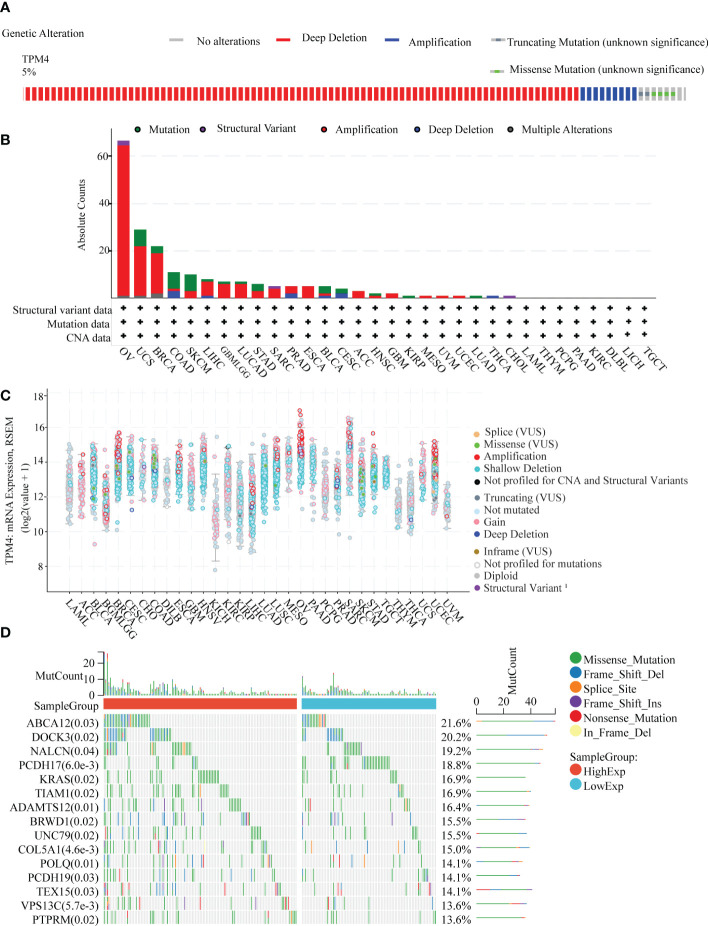
Genetic alteration analysis. **(A)** Genetic alteration in TPM4 in pan-cancer tissues, accounting for 5% of alterations (altered/profiled = 131/2565). **(B)** The alteration frequency with the mutation type of TPM4 in different cancers **(C)**. The mRNA expression of TPM4 putative copy-number alteration (CAN) in pan-cancer tissues **(D)**. The top 15 genes with the highest frequency of mutations in the high TPM4 expression group and low TPM4 expression group in STAD.

We investigated the pan-cancer genetic alterations in TPM4 across the cBioPortal. As shown in **Figure 4A**, TPM4 expression was altered in 131 samples collected from 2565 patients with different cancer types, which accounted for 5% of the samples. The TPM4 mutation rate was observed in 32 types of cancer, as shown in **Figure 4B**. The highest mutation rate (>60%) was observed in cases with OV. Higher mutation rates were not observed in 24 cancers, including STAD. The most common alterations observed were the "mutation" and "amplification" types in copy number variation (CNV) in STAD. Following this, as shown in **Figure 4C**, we investigated the correlation between putative copy-number alteration (CNA) in TPM4 and TPM4 mRNA expression in pan-cancer tissues. Fifteen mutated genes were identified within the mutation spectrum of TPM4 high/low expression cohorts in STAD. The top 5 genes were ABCA12, DOCK3, NALCN, PCDH17, and KRAS (**Figure 4D**).

### Epigenetic alteration analysis

**Figure 5:**
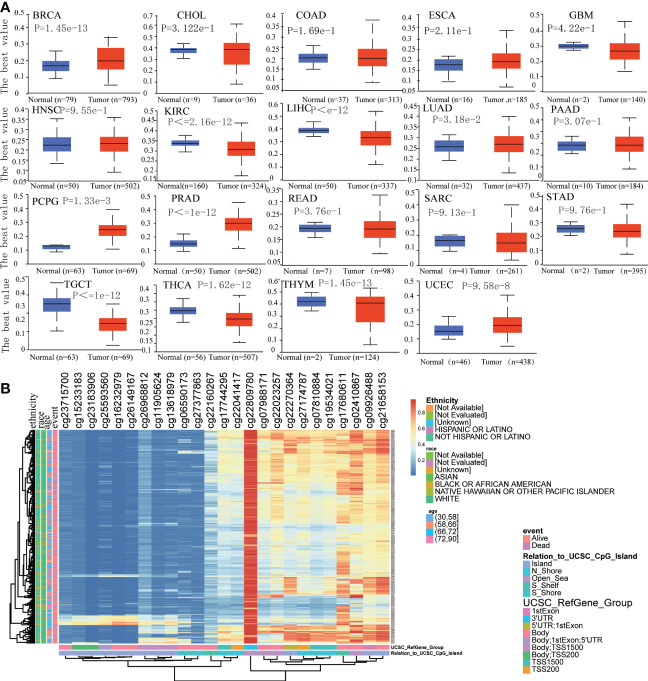
Analysis of DNA methylation in TPM4. **(A)**. Promoter methylation level in TPM4 between normal and tumor tissues in 19 types of cancer from UALCAN **(B)**. The heatmap of TPM4 DNA methylation in STAD from MethSurv.

**Figure 6:**
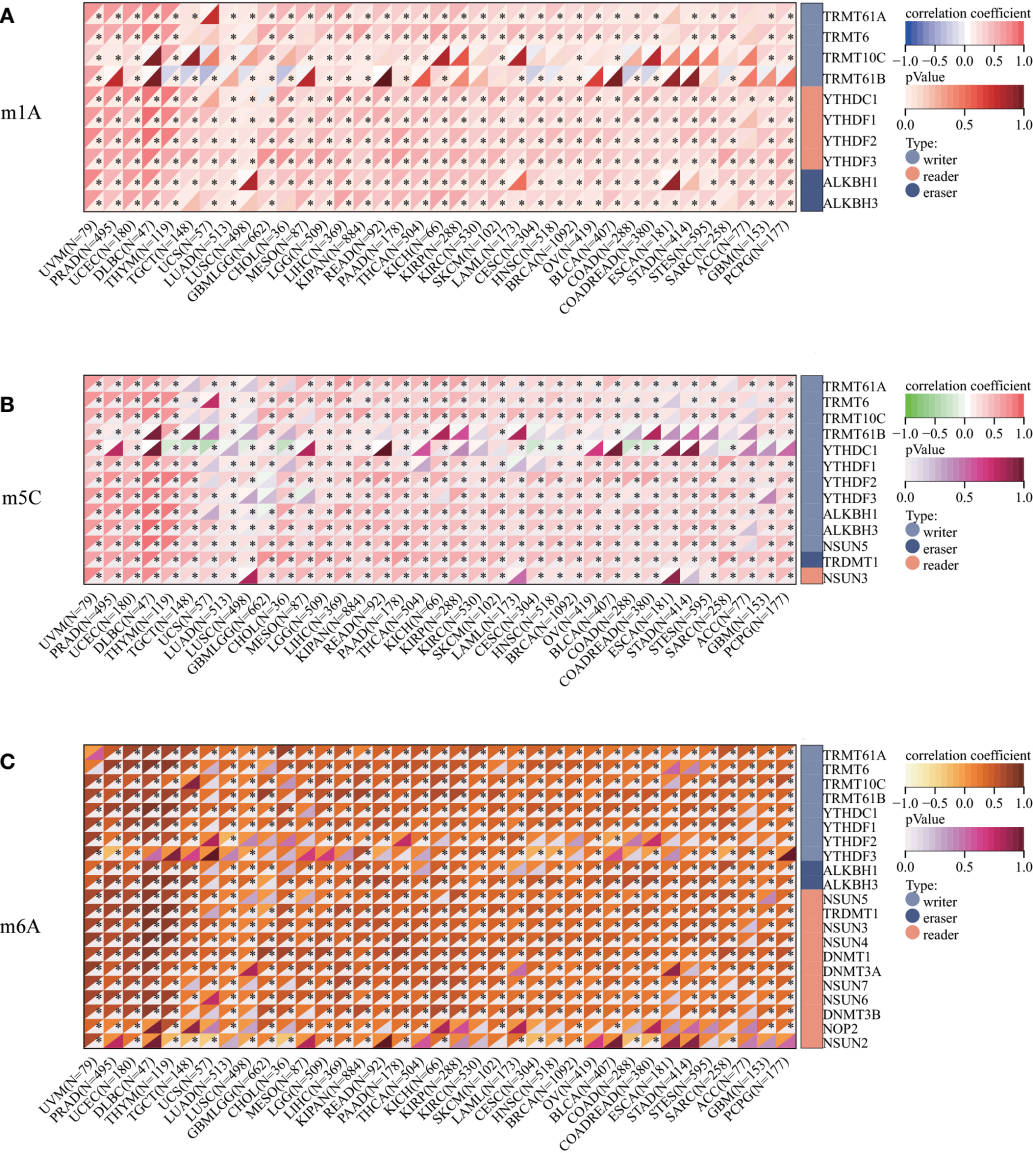
Correlation analysis between TPM4 expression and mRNA modification methylation regulatory factors. **(A)** m1A, **(B)** m5C, **(C)** m6A. Correlations depend on Pearson's rho values and statistical significance. (*p < 0.05).

We assessed the promoter DNA methylation levels in TPM4 in normal tissues and 19 types of cancer tissues. TPM4 was hypermethylated in BRCA, CHOL COAD, ESCA, HNSC, LUAD, PCPG, PRAD, THYM, and UCEC. On the contrary, it was hypomethylated in GBM, KIRC, LICH, PAAD, SARC, STAD, TGCT, and THCA (**Figure 5A**). Following this, according to the methylation map of TPM4 from the MethSurv database, 25 CpG sites of TPM4 were identified in STAD (**Figure 5B** and **Supplementary Table 4**). In addition, the mRNA modification parameter is a crucial component of epigenetics and is involved in post-transcriptional gene regulation. Many studies have shown that mRNA modification is closely related to cancer progression and incidence (33). m1A, m5C, and m6A are common types of mRNA modification. To explore the correlation between TPM4 expression and 45 mRNA modification regulators (see **Supplementary Table 5**), methyltransferases (writers), demethylases (erasers), and RNA-binding proteins (readers) were selected. As shown in **Figures 6A–C**, TPM4 expression was positively related to most m1A, m5C, and m6A methylations in pan-cancer tissues. Subsequently, in STAD, the top 10 methylation regulatory factors included METTL14 (m6A_writer; r=0.403, P < 0.0001), YTHDF3 (m1A_reader; r=0.421, P < 0.0001), FTO (m6A_eraser; r=0.399), TET2 (m5C_eraser; r=0.375), NSUN3 (m5C_writer; r=0.374), WTAP (m6A_writer; r= 0.368), YTHDC2 (m6A_reader; r=0.366), CBLL1 (m6A_reader; r=0.357), FMR1 (m6A_reader; r= 0.338), and YTHDC1 (m1A_reader; r= 0.306). The above outcomes suggest that TPM4 expression is closely associated with DNA methylation and mRNA modification in different cancers, including STAD.

### TPM4 expression and immune infiltration

**Figure 7:**
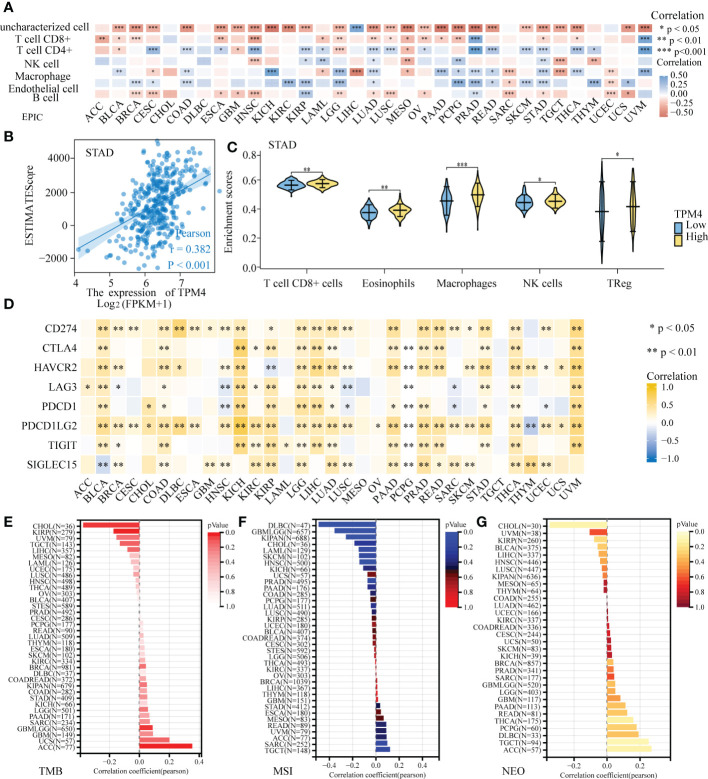
Correlation analysis of TPM4 expression and immune infiltration. **(A)** TPM4 expression and immune cell infiltration. **(B)** The relationship between TPM4 mRNA expression and ESTIMATE scores in STAD. **(C)**. Enrichment scores of TPM4 high and low expression groups in different immune cells of STAD. **(D)** Correlation analysis of TPM4 expression and immune checkpoints in pan-cancer tissues. **(E-G)** Correlation of TPM4 expression and tumor mutation burden (TMB, E) microsatellite instability (MSI, F), and neoantigen expression (NEO, G) in pan-cancer tissues. (*P<0.05, **P<0.01, ***P<0.001).

We investigated the association between TPM4 expression and immune cell infiltration within the tumor microenvironment (TME). The EPIC online tool showed that eight cancer-associated immune cells were related to TPM4 expression in different cancers, especially in KIPR, LUAD, PCPG, PRAG, STAD, and UVM (**Figure 7A**). The ESTIMATE score is useful for determining tumor purity and immune cell infiltration within the TME. Our findings revealed that TPM4 expression is positively related to the ESTIMATE score in STAD (**Figure 7B**). Next, we assessed the enrichment scores of TPM4 high- and low-expression cohorts in immune cells, including CD8 T+ cells, eosinophils, macrophages, NK, and Treg cells in STAD. The enrichment scores within the two cohorts showed significant differences (**Figure 7C**). The immune checkpoint (ICP) gene was found to play a role in immune cell infiltration and immunotherapy outcomes (34). Our result indicates that TPM4 expression was positively related to the expression of these eight ICP genes (CD274, CTLA4, HAVCR2, LAG3, PDCD1, PDCD1, LG2, and TIGIT) in BRCA, CHOL, COAD, DLBC, ESCA, KICH, LGG, LIHC, LUAD, PAAD, PRAD, READ, STAD, THCA, and UVM (**Figure 7D** and **Supplementary Table 6**). This suggests that TPM4 coordinates ICP gene activity through different signaling pathways and may be a pivotal target for immunotherapy. TMB, MSI, and NEO are considered predictors for response to tumor immunotherapy within the TME (35–37). Moreover, we observed the increased expression of TPM4 and the consequent increase within the TMB in ACC, UCSC, GBM, PAAD, and STAD (**Figure 7E** and **Supplementary Table 7**). MSI showed a positive association with TPM4 expression in TGCT, SARC, ACC, UVM, and STAD (**Figure 7F** and **Supplementary Table 8**). TPM4 expression was positively associated with NEO in ACC, TGTC, DLBC, PCPG, and THCA (**Figure 7G** and **Supplementary Table 9**). Collectively, TPM4 may affect antitumor immunity through its association with immune infiltrating cells, ICPS, MSI, TMB, and NEO in pan-cancer tissues.

### lncRNAs-miRNA-TPM4 network construction in STAD

**Figure 8:**
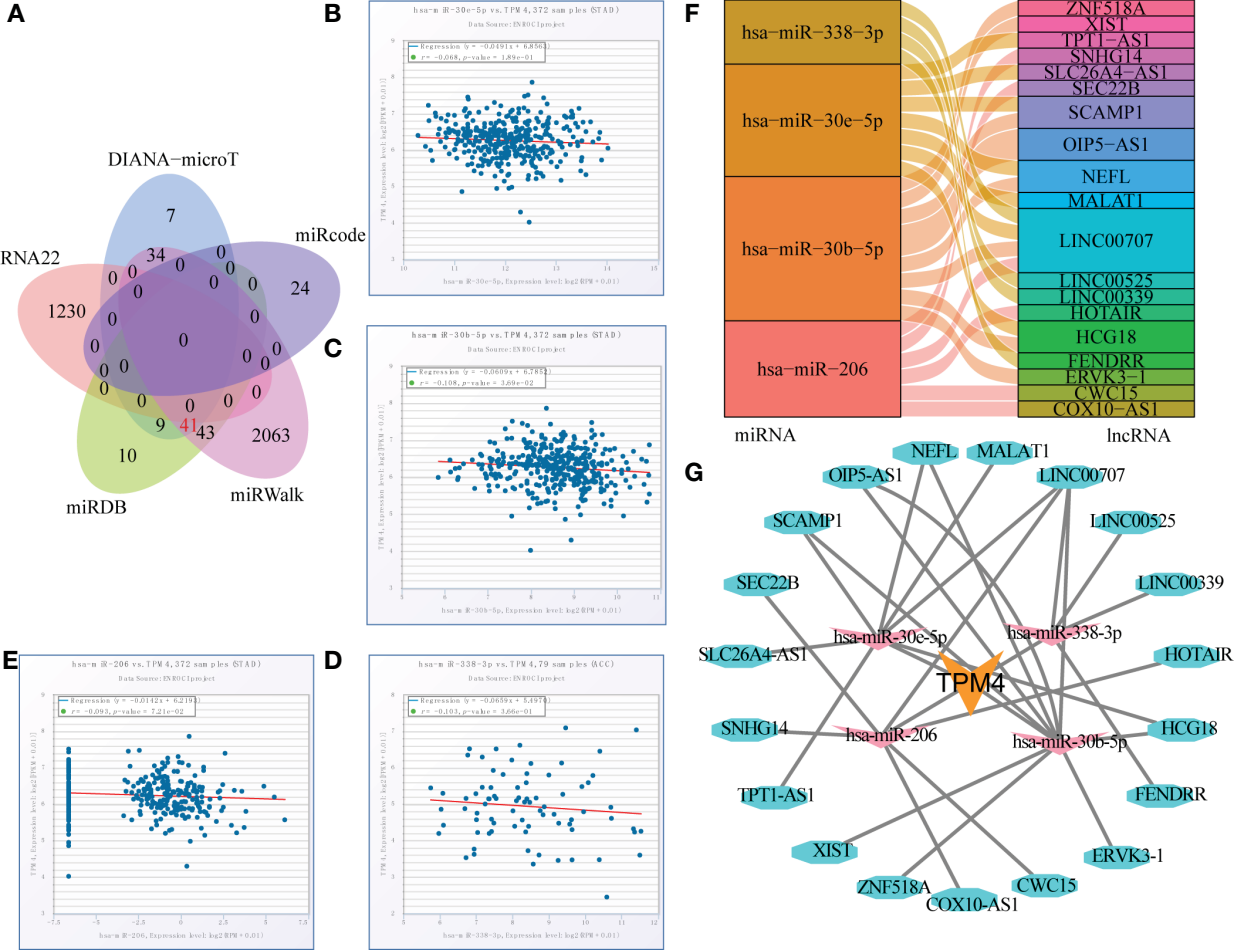
TPM4-related pivotal ceRNA network construction in STAD. **(A)** Venn diagram of predicted TPM4 target miRNAs in RNA22, DIANA-mircoT, miRWalk, miRcode, and TargetScan. **(B-E)** Correlation analysis of TPPM4 and the target miRNAs in scatter plots. hsa -miR-30e-5P **(B)**, hsa -miR-30b-5p **(C)**, hsa -miR-338-3P **(D)**, and hsa -miR-206 **(E)**. **(F)** Sankey diagram of target miRNAs and their target lncRNAs. **(G)** The lncRNA-miRNA-TPM4 regulatory network was constructed for STAD using Cytoscape.

RNA22, DIANA-micro, miRWalk, miRcode, and TargetScan were used to identify the target miRNAs of TPM4 in STAD, as shown in **Figure 8A** and **Supplementary Table 10**. We identified 7, 24, 2063,10, and 1230 TPM4 target miRNAs from these sources, respectively. Forty-one common miRNAs were predicted in three databases. To further explore the target lncRNAs of 41 miRNAs, Starbase2.0 was used, and seven miRNAs (hsa -miR-613, hsa -miR-338-3P, hsa -miR-206, hsa -miR-30e-5P, hsa -miR-30b-5p, hsa -miR-10b-5p, and hsa -miR-299-3p) showed target lncRNAs. mRNA expression is generally negatively correlated with miRNA expression (38). TPM4 expression was negatively correlated with hsa-miR-30e-5P (**Figure 8B**), hsa -miR-30b-5P (**Figure 8C**), hsa s-miR-338-3 (**Figure 8D**), and hsa -miR-206 expression (**Figure 8E**). lncRNAs could act as competitive endogenous miRNAs to further regulate mRNA expression (39). We also identified four target miRNAs and their target lncRNAs that were regulated in a negative manner (**Figure 8F** and **Supplementary Table 11**). Finally, we constructed a lncRNAs-miRNAs-TPM4 regulatory network for GC (**Figure 8G**).

### Pan-cancer sensitivity of TPM4-related drugs

**Figure 9:**
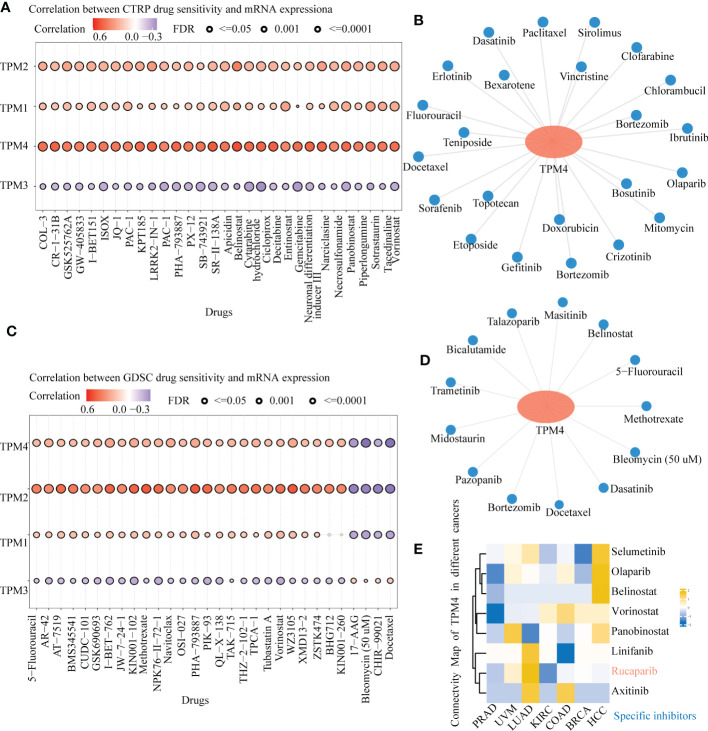
Drug sensitivity of TPM4-related drugs in pan-cancer tissues. **(A)**. Relationship between CTRP drug sensitivity and mRNA expression of TPM1, TPM2, TPM3, and TPM4. **(B)** FDA-approved TPM4-related chemotherapeutic drugs from CTRP drug sensitivity **(C)**. Correlation between GDSC drug sensitivity and mRNA expression of TPM1, TPM2, TPM3, and TPM4. **(D)** FDA-approved TPM4-related anti-cancer drugs from GDSC drug sensitivity. **(E)** Heatmap of small molecules drugs targeting in TPM4 from Connective map(CMap).

The CTPR dataset indicated the correlation between members of the TPM family (TPM1, TPM2, TPM3, TPM4) mRNA expression levels and drug sensitivity; the top three drugs that were positively related to TPM4 expression were COL-3 (incyclinide), CR-1-31-B (eIF4A inhibitor), and GSK525762A (Bet inhibitor); (**Figure 9A** and **Supplementary Table 12**; P < 0.0001). Based on GDSC drug sensitivity outcomes, the top three drugs that were positively related to TPM4 expression were 5-fluorouracil, AR-429 (histone deacetylase inhibitor), and AT-7519 (inhibitor of cyclin-dependent kinases), and the ones that were negatively related to TPM4 expression were 17-AAG (inhibitor of heat shock protein 90), bleomycin (50 uM), CHIR -99021 (GSK-3 α/β inhibitor), docetaxel (**Figure 9C** and **Supplementary Table 14**, P < 0.0001). As some drugs that feature within the prediction outcomes for CTRP and GDSC drug sensitivity are used in scientific research, 23 (**Figure 9B** and **Supplementary Table 13**) and 13 (**Figure 9D** and **Supplementary Table 15**) types of TPM4-related antitumor drugs approved by the FDA are based on data from drug banks. We analyzed DEGs with high and low expression of TPM4 by CMap “qury” online tool. Based on the results of CMap database inquiry, 15 types of small molecules drugs including ALK/ BCR-ABL/ BTK/ CDK /Met inhibitor, etc. were identified (**Supplementary Table 16**), eight small molecular drugs targeting TPM4 obtained (**Supplementary Table 16**), meaning that they have the potential to treat PRAD. UVM, LUAD, KIRC, COAD, BRCA, HCC. Notably, Rucaparib had the highest absolute value score, meaning that the drug has the potential to treat the7 types of cancer (**Figure 9E**). Rucaparib (40) is a poly (ADP-ribose) polymerase (PARP) inhibitor used to treat recurrent ovarian and prostate cancers.

### Genes exhibiting relative co-expression, and functional analysis of TPM4 expression in STAD

**Figure 10:**
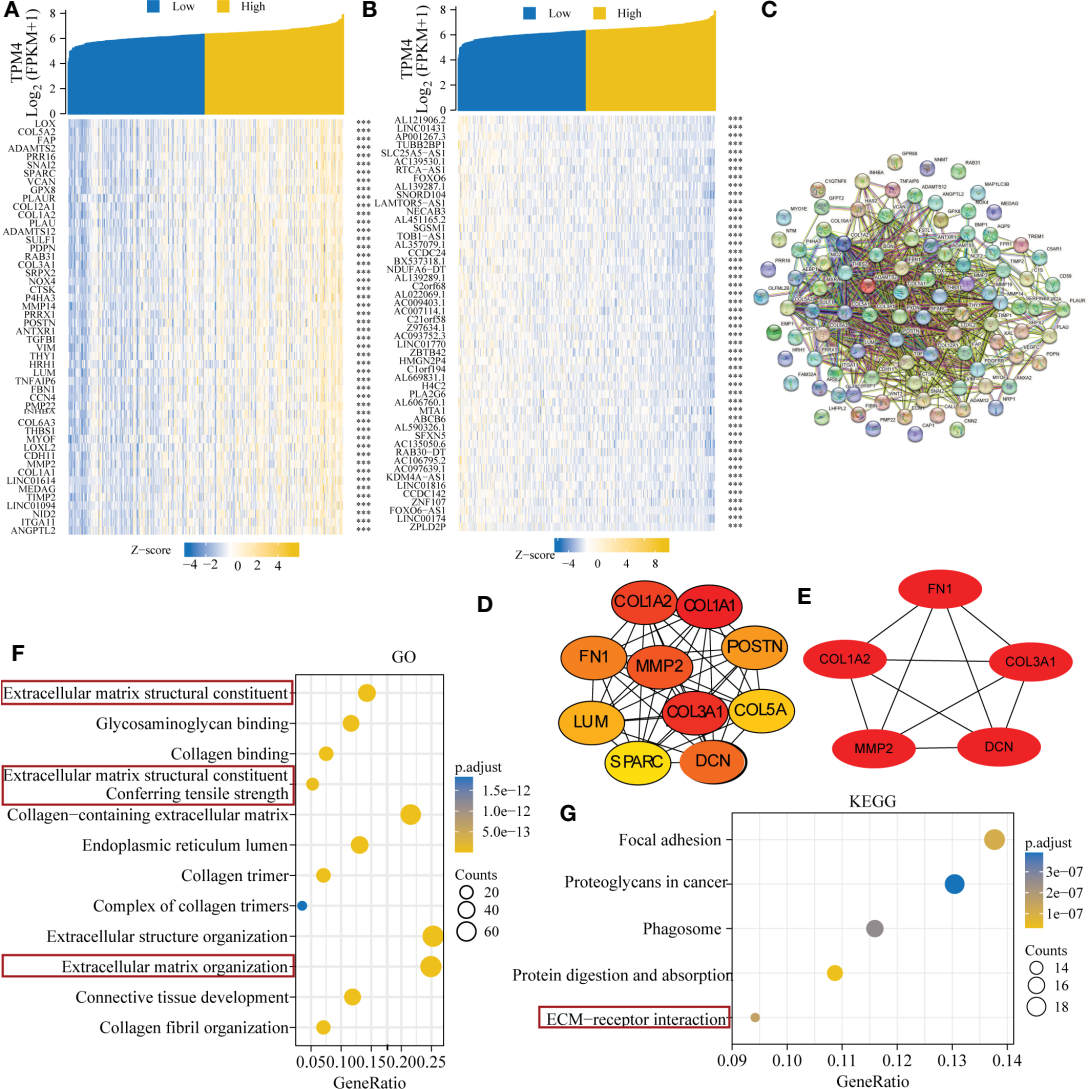
Related genes showing co-expression, and functional analysis of TPM4 expression in STAD. **(A-B)** Expression heatmap of top 50 co-expressed genes positively and negatively correlated with TPM4 expression in STAD. **(C)** The TOP 100 co-expressed genes positively related to TPM4 expression are shown in the PPI network across STRING. **(D, E)** The top 10 and top 5 hub genes of the network and the MCODE2 components were identified. **(F, G)** Gene Oncology (GO) and Kyoto Encyclopedia of Genes and Genomes (KEGG) analysis of TPM4 and 300 co-expressed genes.

We identified genes exhibiting co-expression from TCGA. The heatmap shows the top 50 co-expressed genes with their expression positively and negatively correlated with TPM4 expression in STAD (**Figures 10A, B**; **Supplementary Table 17**). The top 100 co-expressed genes with their expression positively related to TPM4 expression are shown in the PPI network (**Figure 10C**; **Supplementary Table 17**). The top 10 hub genes were COL1A2, COL1A1, CLO3A1, COL5A, POSTN, FN1, MMP2, LUM, SPARC, and DCN (**Figure 10D**). The top 5 hub genes were COL1A2, CLO3A1, FN1, MMP2, LUM, SPARC, and DCN (**Figure 10E**). Following this, GO and KEGG enrichment analyses were conducted using the top 300 co-expressed genes. The GO analysis involved molecular function, cellular components, and biological processes. (See **Figure 10F** and **Table 1**). KEGG pathway enrichment (See **Figure 10G** and **Table 2**). The outcomes revealed that TPM4 expression plays a role in GC by regulating the extracellular matrix.

**Table 1:** Gene Ontology (GO) analyses of the top 300 co-expression genes positively associated with TPM4 expression.

ONTOLOGY	ID	Description	GeneRatio	BgRatio	pvalue	p.adjust
BP	GO:0030198	extracellular matrix organization	67/269	368/18670	8.36e-55	2.93e-51
BP	GO:0043062	extracellular structure organization	68/269	422/18670	6.21e-52	1.09e-48
BP	GO:0030199	collagen fibril organization	19/269	54/18670	6.37e-22	7.44e-19
BP	GO:0061448	connective tissue development	32/269	273/18670	4.61e-20	4.04e-17
BP	GO:0051216	cartilage development	28/269	209/18670	3.05e-19	2.14e-16
CC	GO:0062023	collagen-containing extracellular matrix	61/283	406/19717	1.76e-44	4.92e-42
CC	GO:0005788	endoplasmic reticulum lumen	37/283	309/19717	2.05e-23	2.87e-21
CC	GO:0005581	collagen trimer	20/283	87/19717	7.04e-19	6.57e-17
CC	GO:0098644	complex of collagen trimers	10/283	19/19717	2.61e-14	1.83e-12
CC	GO:0044420	extracellular matrix component	13/283	51/19717	2.44e-13	1.04e-11
MF	GO:0005201	extracellular matrix structural constituent	38/266	163/17697	1.43e-34	5.69e-32
MF	GO:0005518	collagen binding	20/266	67/17697	5.21e-21	1.03e-18
MF	GO:0005539	glycosaminoglycan binding	31/266	229/17697	8.02e-21	1.06e-18
MF	GO:0030020	extracellular matrix structural constituent conferring tensile strength	14/266	41/17697	5.23e-16	5.20e-14
MF	GO:0050840	extracellular matrix binding	15/266	57/17697	3.82e-15	3.03e-13

**Table 2:** Kyoto Encyclopedia of Genes and Genome enrichment (KEGG) analyses of the top 300 co-expression genes positively associated with TPM4 expression.

ONTOLOGY	ID	Description	GeneRatio	BgRatio	pvalue	p.adjust
KEGG	hsa04974	Protein digestion and absorption	15/138	103/8076	1.63e-10	3.19e-08
KEGG	hsa04510	Focal adhesion	19/138	201/8076	1.09e-09	1.06e-07
KEGG	hsa04512	ECM-receptor interaction	13/138	88/8076	2.41e-09	1.58e-07
KEGG	hsa04145	Phagosome	16/138	152/8076	5.24e-09	2.57e-07
KEGG	hsa05205	Proteoglycans in cancer	18/138	205/8076	1.01e-08	3.92e-07

### TPM4 knockdown inhibits cell migration and tumor invasion

**Figure 11:**
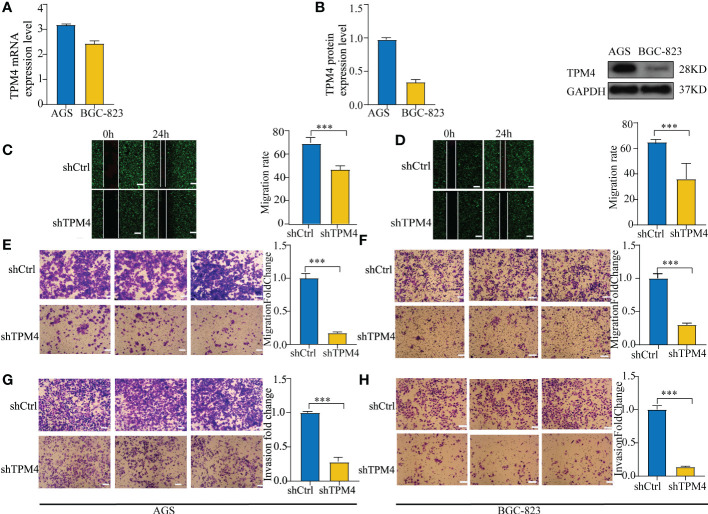
Knockdown of TPM4 inhibits migration and invasion. **(A, B)**. The mRNA **(A)** and protein expression **(B)** levels of TPM4 in AGS and BGC-823 cell lines. **(C-F)** Wound healing and transwell assays for assessing the migration potential of AGS **(C, E)** and BGC-823 **(D, F)** cells with shCtrl/shTPM4 after TPM4 knockdown. **(G, H)** Matrigel transwell assays were used to measure the invasive potential of AGS **(G)** and BGC-823 **(H)** cells transfected with shCtrl/shTPM4.( ***p< 0.001, compared with shCtrl.).

To verify the function of TPM4 in GC, we used two types of GC cells in our laboratory. The data showed the mRNA (**Figure 11A**) and protein expression (**Figure 11B**) levels of TPM4. In our previous study, after shRNA lentivirus infection, the knockdown efficiency of shTPM4 was 71.1%, and the knockdown of TPM4 inhibited GC cell proliferation (15) (**Supplementary Figure 2**). In this research, wound healing and transwell assays were used to determine the migration potential of GC cells. The knockdown of TPM4 repressed the migration of AGS (**Figure 11C**, P < 0.01) and BGC-823 (**Figure 11D**, P< 0.01), as observed in the wound healing assay. We also found that TPM4 expression inhibited the migration of AGS cells (**Figure 11E**, P < 0.01) and BGC-823 cells (**Figure 11F**, P < 0.01). The Matrigel transwell assay showed that the invasive potential was significantly reduced in AGS cells (**Figure 11G**, P < 0.01) (G) and BGC-823 cells (**Figure 11H**, < 0.01) with TPM4 knockdown. The outcomes of these experiments showed that TPM4 is a key oncogene that promotes tumor invasion and cell migration in GC.

## Discussion

GC is one of the most common malignant gastrointestinal tumors. The prevalence of early diagnosis and optimization of treatment strategies have led to a downward trend in incidence and mortality. However, the prognosis is still poor, with a 5-year survival rate of less than 20%, especially for metastatic GC, which has a survival rate of less than 1 year (1). We analyzed the expression, diagnosis, prognosis, and immune infiltration of a meaningful biomarker in pan-cancer tissues and explored the regulatory network of ceRNA in GC, drug sensitivity, and molecular function, combined with molecular experiments for validation. Our findings provide a new perspective on the pathogenesis of GC and clinical treatment strategies.

TPM4 is an actin-binding protein that is associated with the development of different tumors, including STAD, LICH, LUAD, HCC, and BRCA. However, an integrated bioinformatics analysis of the function of TPM4 in pan-cancer tissues across multiple databases has not been performed to date. In our study, TPM4 expression was upregulated in most cancer tissues compared to that in normal paired tissues. TPM4 expression was found to be lower express in BLCA, KICH, KIRC, KIRP, UCEC, and PRAD. There is no relevant literature reporting the relationship between TPM4 expression and these tumors with TPM4 down-regulated. This is indeed an interesting topic, further experimental verification is necessary to elucidate the biological functions of TPM4 in these cancers., we intend to perform more careful analyses of the diagnosis and treatment effects of these predicted TPM4 downregulated in the cancers by combining *in vivo* and *in vitro* techniques. We intend to analyze the basic expression patterns and clinical value of the most significant.TPM4 protein had the highest diagnostic efficiency, and patients with low TPM4 expression showed a good prognosis in PAAD, consistent with previous outcomes (14). In addition, TPM4 expression was confirmed to be upregulated in GC tissues (N=50) and HCC tissues compared with that in adjacent tissues (15)) and was associated positively with the T status, grade, and stage of HCC (N=10) (41). These outcomes indicate that TPM4 is a prospective biomarker of diagnosis and prognosis in different cancers, including GC.

The mutation is caused by the transformation of gene sequence, which affects the development of tumors (42). Unlike mutations, epigenetic alterations do not alter the primary DNA sequence (43). However, mutation and epigenetic alterations lead to the same consequence: abnormal gene expression. Following this, 15 genes, including ABCA12, DOCK3, NALCN, PCDH17, and KRAS, were found to exhibit high mutation frequencies within the TPM4 high- and low-expression cohorts of STAD. Epigenetic alterations, such as DNA methylation, miRNA modification, and tumor development, involve the activation and inactivation of oncogenes. Since DNA methylation of the CpG islands within the gene promoter region can cause the silencing of gene expression (44), we assessed the promoter DNA methylation levels of TPM4 in 19 types of cancers and normal tissues. TPM4 is hypomethylated in different cancers, including STAD, and 25 CpG sites of TPM4 were observed in STAD. Furthermore, our study showed that in most cancers, including STAD, the expression of TPM4 mRNA exhibited positive correlation with a majority of m1A, m5C, and m6A methylation regulators. Therefore, TPM4 could be used as a potential diagnostic marker for detecting mutations and pan-cancer epigenetic changes.

Human immunotherapies have yielded novel treatment strategies for patients with cancer and drastically altered the landscape of oncology. However, not every patient can show effective outcomes from immunotherapy and maintain a long-term clinical response. TME heterogeneity may lead to some patients not benefiting from treatment. In our study, TPM4 expression was related to immune cell infiltration in KIPR, LUAD, PCPG, PRAD, STAD, and UVM. This revealed that TPM4 can affect a patient’s sensitivity to immunotherapy and may be a therapeutic target worth exploring for these tumors. TPM4 expression was correlated with the expression of the majority of ICP genes as well as MSI, TMB, and NEO in multiple cancers. Therefore, TPM4 may be a prospective biomarker for the immunotherapeutic response in patients at a pan-cancer level, including STAD. Notably, the ESTIMATE score supports the association between the expression of TPM4 and the infiltration of immune cells. There were differences in expression within the high- and low-TPM4 expression cohorts in CD8+ T cells, eosinophils, macrophages, NK cells, and Treg cells. TPM4 expression is related to MSI and TMB, which can be of value in further investigations on TPM4 expression in STAD immunotherapy.

The competitive binding of lncRNA with miRNA affects mRNA expression. The lncRNA-miRNA-mRNA regulatory mechanism is evident in STAD. miR-206 inhibits GC proliferation (45). LINC00707 promotes GC cell proliferation and metastasis (46). HOTAIR knockdown exerts an anti-tumor effect by stimulating miRNA-206 expression to inhibit the expression of CCND1 and CCND2 (47). The network lncRNA-miRNA-TPM4 was developed based on the miRNAs hsa-miR-338-3p, hsa-miR-30e-5p, hsa-miR-30b-5p, and hsa-miR-206, along with LINC00707, HOTAIR, and seventeen other lncRNAs. Here, The ceRNA network of TPM4 was identified to reveal the mechanism underlying GC progression.

We investigated the sensitivity of TPM4-related drugs. We identified 23 and 13 types of chemotherapeutic drugs related to TPM4 expression from CTRP and GDSC, respectively. 5-fluorouracil and docetaxel are first-line and second-line chemotherapeutic drugs (1) of GC, respectively. TPM4 expression was found to be related to the drug sensitivity of docetaxel and 5-fluorouracil. TPM4 may be a marker predicting the treatment effect of docetaxel and 5-fluorouracil. From CMap dataset, eight small molecule drugs (**Figure 9E**) were identified with the potential to treat PRAD. UVM, LUAD, KIRC, COAD, BRCA, HCC. Notably, Rucaparib had the highest absolute value score, meaning that the drug has the most potential to treat the 7 types of cancer. However, the underlying molecular mechanism needs further investigation.

The extracellular matrix (ECM) is a structure that forms the mesenchymal and epithelial vasculature matrix, which comprises structural protein, connexin protein, polysaccharide-protein, and secretory proteins It interacts with cells to deliver extracellular signals (48). Multiple cellular receptors connect with the components of the ECM (49). Interactions with the ECM and cell surface receptors regulate cell behavior and act as critical functions in communication between cells, cell proliferation, anoikis (50), adhesion, and migration. We identified five hub genes of TPM4 co-expression, namely collagen 1A (COL1A1), collagen 3A1 (COL3A1), decorin (DCN), fibronectin 1 (FN1), and matrix metallopeptidase 2 (MMP2). COL1A1 (51) and COL3A1 (52) are structural proteins that surround the surrounding non-fibrous components that form the ECM skeleton. DCN (53), which is a polysaccharide-protein of the ECM, binds to cell surface receptors and mediates cancer suppression. FN1 (54) enhances the adhesion between tumor cells and the anchoring between tumor cells, the matrix, and the basement membrane without shedding and metastasis. MMP2 (55) acts as a critical component in the degradation of almost all ECM components, including collagen, proteoglycan, and laminin. ECM remodeling creates a loosely held microenvironment for growth as well as tumor cell differentiation, which promotes a high rate of proliferation, a low rate of differentiation, as well as tumor cell invasion and metastatic spread. GO as well as KEGG analyses were performed on genes that were co-expressed with TPM4 in GC. Focal adhesion, proteoglycans in cancer, phagosome, and pathways for receptor interaction within the ECM were found to be enriched. According to our previous study (15), TPM4 was shown to promote proliferation and inhibit apoptosis in GC cells *in vitro* as well as *in vivo*. In this research, we observed that cell migration and invasion were inhibited when TPM4 was knocked down. We speculated that TPM4 expression influences the specific biological behavior of GC cells through ECM remodeling.

The study had some limitations. TPM4 affects immune infiltration and is related to MSI and TMB as an effective target for immunotherapy, TPM4 is also a target for predicting the drug sensitivity of docetaxel and 5-fluorouracil in GC. However, the application of findings from clinical trials is currently needed. We identified the ceRNA network of TPM4 in GC, which needs further experimental validation. We found that TPM4 stimulated GC proliferation and invasion *in vitro*. The mechanism by which TPM4 affects GC invasion and metastasis *via* ECM remodeling needs to be validated *in vitro* and *in vivo* experiments.

## Conclusions

TPM4 serves as a promising biomarker for prognostic and diagnostic and immunotherapy in cancers, including GC. In addition, TPM4 expression was correlated with docetaxel and 5-fluorouracil sensitivity. The lncRNA-miRNA-TPM4 network regulates the mechanism of GC progression. The function of TPM4 as an oncogene and a promoter of the invasion and migration of GC cells, possibly through ECM remodeling, needs further investigation.

## Data availability statement

The original contributions presented in the study are included in the article/**Supplementary Material**. Further inquiries can be directed to the corresponding author.

## Author contributions

Conceptualization, QG and YSL; Methodology, Formal analysis, Visualization, and Writing original draft, QG; Software, CG, SD, JH; Experiment validation, LZ, NY, and XS; Data collection, QG, LZ, NY, YL, and CG; Project administration and Funding acquisition, YSL and QG. All authors contributed to the article and approved the submitted version.
