# Supplementary figures and images for: Integrated pan-cancer analysis and experimental verification of the roles of tropomyosin 4 in gastric cancer
Source: Front Immunol. 2023 Mar 13;14:1148056. doi: 10.3389/fimmu.2023.1148056 (PMC10041708; doi:10.3389/fimmu.2023.1148056)

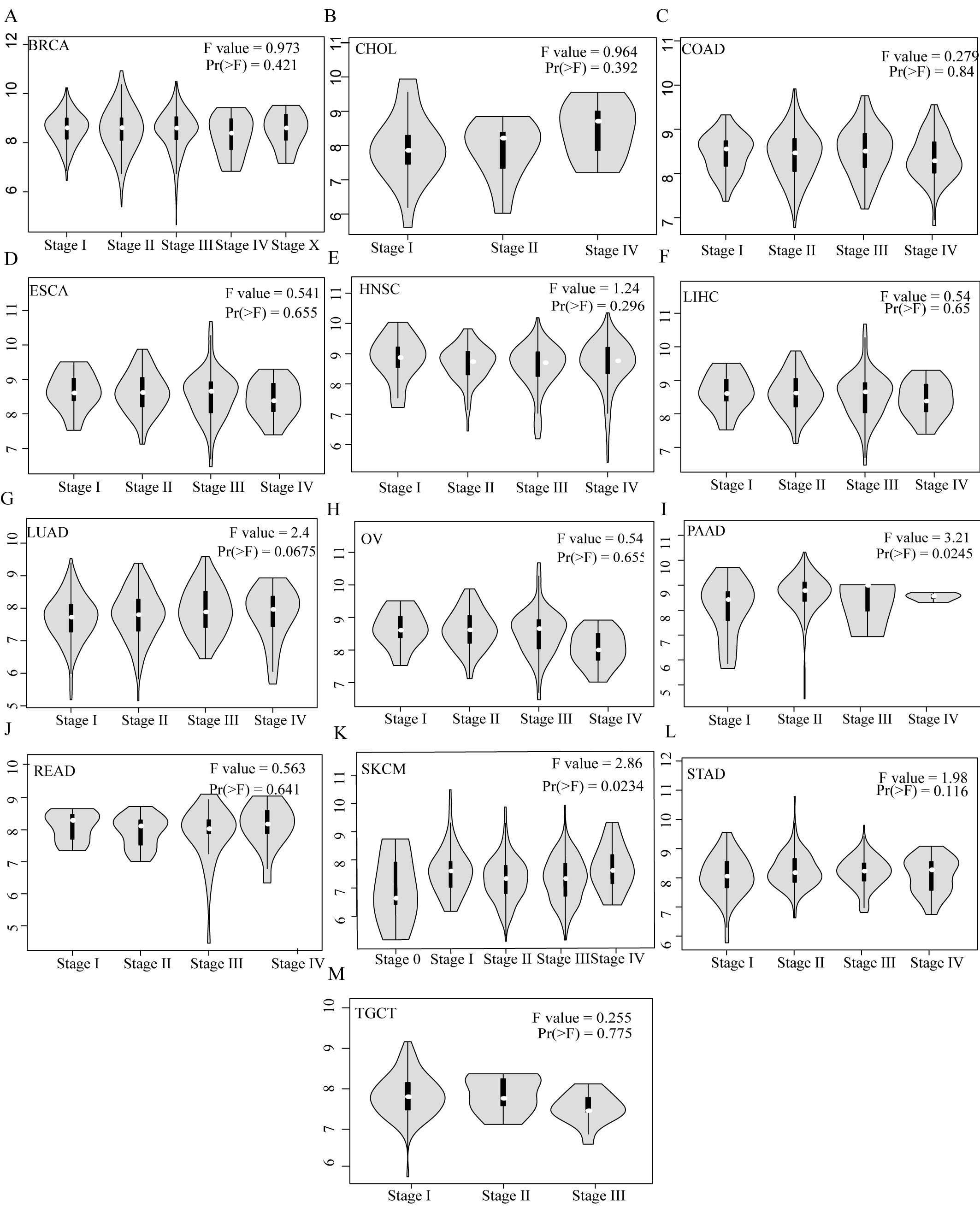

Supplement: Supplementary file 1 [file Image_1.tif]

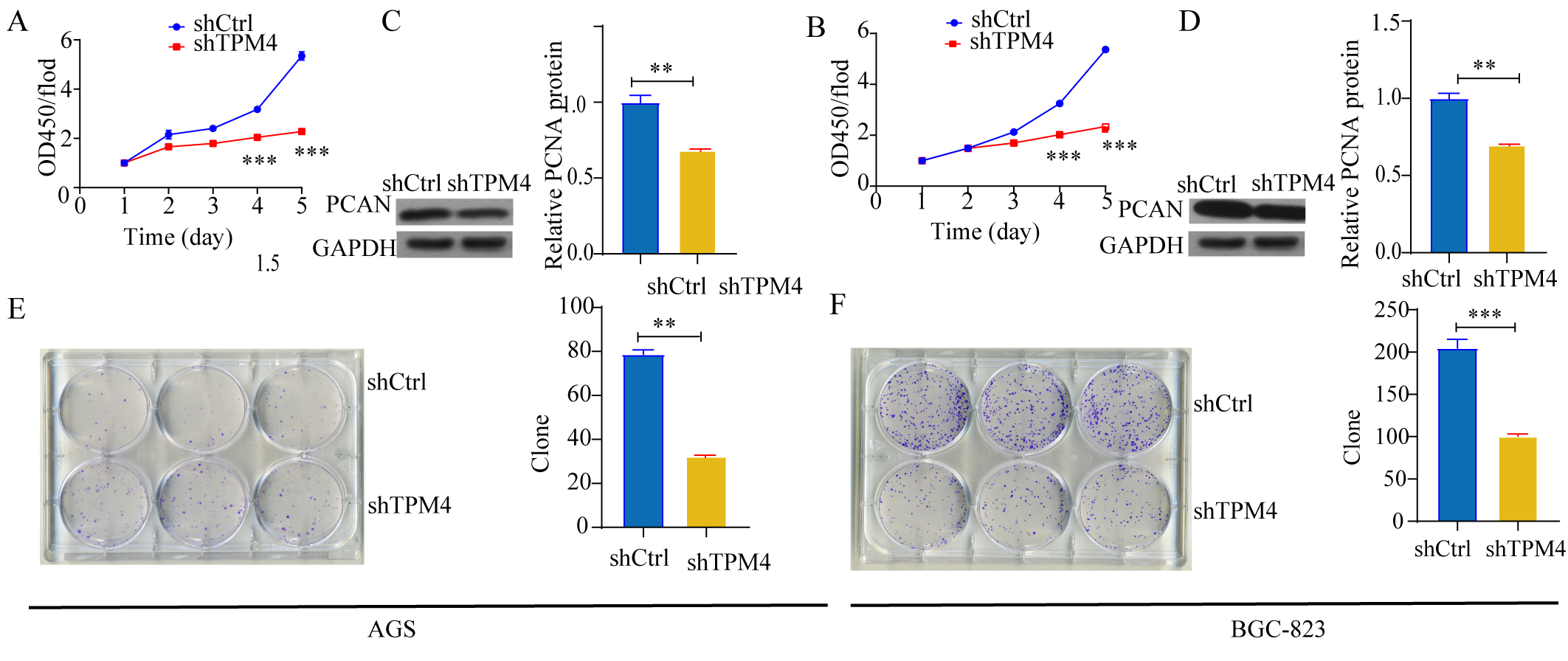

Supplement: Supplementary file 2 [file Image_2.tif]
